# Association of Malnutrition With Weaning Practices Among Infants in Pakistan

**DOI:** 10.7759/cureus.31018

**Published:** 2022-11-02

**Authors:** Sidra Ajmal, Laiba Ajmal, Maleeha Ajmal, Gul Nawaz

**Affiliations:** 1 Chemistry and Biochemistry, University of Oklahoma, Norman, USA; 2 Internal Medicine, Marshfield Clinic Health System, Marshfield, USA

**Keywords:** breastfeeding, vitamin deficiency, infants, weaning practices, malnutrition

## Abstract

Background

An appropriate diet is critical for the growth and development of infants, especially in the first two years of life. Despite considerable efforts made by government and local authorities to raise awareness, mothers still lack basic knowledge of weaning practices; as a result, infants face a growing number of health problems in their later years of life. This research aims to investigate the association between malnutrition and knowledge of different weaning practices among mothers and to study the factors influencing it.

Methodology

The current study was a cross-sectional study conducted at Mayo Hospital, Services Hospital, and Jinnah Hospital, Lahore, Pakistan between November 2019 and May 2020. A total of 200 infants of both genders aged between one and 12 months were included in the study. Knowledge and practices of complementary feeding by the mothers were assessed using a close-ended, pre-tested questionnaire.

Results

Mothers of 200 infants were interviewed and it was found that 79.5% of the infants were being breastfed while 24% never received breast milk. Among those who never received breast milk were given liquids (25%) and semi-solid foods (64.5%). Of the mothers, 8% started weaning at an early age (less than six months old) while the remaining started weaning at the age of six months or later and reported that their infants were either underweight or had reduced heights for that age. Based on the medical reports obtained from the hospital after getting parental consent, it was also found that the infants had distinct signs of deficiency of vitamins A and D, iron, and folate.

Conclusions

The majority of infants were breastfed, and, in most cases, weaning started at an age of less than six months. Most of the infants were fed semi-solid food as their first complementary food. Two-thirds of the infants were underweight for that age, and one-fifth had reduced heights. Based on deficiency statistics, mothers should be encouraged to start weaning at six months to have minimized malnourishment instances in infants.

## Introduction

Malnutrition is an umbrella term that includes but is not limited to undernutrition, obesity, overweight, micronutrient deficiency, etc. [1]. However, it is frequently used only for conditions such as undernutrition from inadequate calories or inadequate provision of specific dietary components. Children often suffer from malnutrition during the age of rapid development, which can have long-lasting impacts on health [2]. Malnutrition is widespread in most developing countries and is particularly prevalent among infants in Africa and Pakistan. The problem is more severe in Pakistan where poverty and illiteracy are predominant.

A balanced diet is critical for the growth and development of infants, especially in the first two years of life. From birth to four months of age, the nutritional needs of all infants are perfectly met by breastfeeding but as the infants reach four to six months, breast milk is no longer sufficient to fully cover their energy and nutrient requirements. This energy and nutrient deficit must be covered by administrating semi-solid food along with breast milk, a process called weaning. Early introduction of weaning has an immediate effect on the health of infants in economically developing countries because of factors such as lack of availability of suitable alternatives to breast milk, microbial contamination of foods and fluids, and displacement of breast milk by less nutritious alternatives. On the contrary, the late introduction of supplementary food can trigger dietary disturbances leading to malnutrition, as indicated by studies in India that late weaning is directly responsible for stunted growth [3]. The World Health Organization (WHO) recommends that infants should get complementary foods at six months of age in addition to breast milk, initially two to three times a day between six and eight months [4].

With that being said, adequate complementary feeding is dependent on having accurate information, family support, and a well-established healthcare system. In the last few decades or so, significant advancements have been made to promote breastfeeding, but unfortunately not for complementary feeding or weaning. Previous studies only focused on limited aspects of weaning such as either the time or frequency of weaning or the consistency of mothers practicing complementary feeding. Considering these limitations, this study was designed to investigate the relationship between malpractices in weaning and malnutrition in infants.

## Materials and methods

The current study was a cross-sectional study conducted at Mayo Hospital, Services Hospital, and Jinnah Hospital, Lahore, Pakistan between November 2019 and May 2020. A total of 200 infants of both genders aged between one and 12 months were included in the study. Premature or infants with any underlying congenital disease were excluded. Those fulfilling the requirements of inclusion and exclusion criteria were recruited for the study from the indoor and outpatient department (OPD) of the pediatrics department. Written informed consent was first taken from the parents of individual infants. Questions included information about demographics, weaning practices, and the age of the infant at which regular weaning was initiated, specifically if the weaning practice started prior to reaching the age of six months or later than six. Additionally, we recorded only two major indicators of nutritional status in infancy: weight and height of the infants.

To do so, we followed the WHO's child growth standards, and each infant was weighed in kilograms by utilizing a standard pediatric weighing machine available at the pediatric departments [5]. Likewise, we measured the infant’s height in centimeters according to the provided standard procedure. With this information, we compared both weight and height with the WHO’s standard percentile growth charts of weight-for-age and height-for-age, respectively. Children having a weight lower than the 50th percentile on the weight-for-age growth chart were categorized as underweight and infants having a height lesser than the 50th percentile on the height-for-age growth chart were categorized as shorter as compared to the normal range. Underweight infants shorter than normal for their specific age for both parameters were rendered as “malnourished.” Data were analyzed via SPSS version 22 (IBM Corp., Armonk, NY). Variables of interest were age, gender, malnutrition, time of weaning, and contents of weaning. Mean values were calculated for numerical variables and frequency tables were generated for nominal variables. Cross tabulation was done with age, gender, and starting time of weaning with dependent variables, i.e., degree and prevalence of malnutrition.

## Results

The sociodemographic characteristics of the participants and infants are shown in Table 1. The results of this study showed that 79.5% of infants were being breastfed while 20.5% were not being breastfed at the time of the study. In the current study, we also found that 64.5% of infants were introduced to semi-solid foods, 25% to liquids, and the remaining 10.5% to solids.

**Table 1:** Sociodemographic profile of participants and status of breastfeeding among infants

Characteristics	Total (N = 200)
Study location (%)	
Outpatient department (OPD)	62
Indoor	38
Education status (%)	
Illiterate	44
Primary	27
High school	17.5
Graduate or above	11.5
Family size (%)	
<4	12.5
5-8	80
9-11	7.5
Child age (%)	
1-6 months	53
7-12 months	47
Gender (%)	
Male	51
Female	49
Breast-fed children (%)	
Yes	79.5
No	20.5
Age of weaning (%)	
<6 months	8
6 months	35.5
>6 months	56.1
Breastfeeding history (%)	
Exclusively	42
Partially	34
Supplementary	24
Weaning food (%)	
Liquid	25
Semi-liquid	64.5
Solids	10.5
Food preferences (%)	
Home-made	68.5
Commercially canned	31.5

The age at which weaning is started in an infant is crucial as starting too soon or introducing solids late can result in the child becoming underweight. Based on the results, a significant association was found between weaning age and underweight infants (Table 2).

**Table 2:** Association of variables with the age of weaning

	Underweight	
Weaning age	Yes	No
>6 months	51.3%	48.7%
At 6 months	32.4%	67.6%
<6 months	62.5%	37.5%
P-value = 0.02		

Of the infants in this study, 44% had a history of suffering from prolonged illnesses when being infected with pneumonia, common cold, etc. (greater than two weeks) at some point in their life (Table 3). Lastly, based only on the medical history obtained from the stored patient’s history at the pediatrics department and not on experimental tests, vitamin A and vitamin D deficiencies were found in the observed infants (see Appendix). Since vitamin A and D deficiency is endemic to Pakistan, macronutrient deficiency observed in infants is a clear indication of maternal deficiencies as well.

**Table 3:** Practices of participants regarding weaning

Characteristics	Total (N = 200)
Illness for <2 weeks (%)	
Yes	44
No	56
Underweight (%)	
Yes	55
No	44
Stunted growth (%)	
Yes	26
No	74
Diarrhea after weaning (%)	
Yes	12
No	88

## Discussion

The results from the socio-demographic analysis revealed a substandard level of knowledge and practice among mothers for weaning. The results correspond to a study in which 40% of infants were breastfed after birth, 89% were breastfed at one point in their lives, and 20.1% were never breastfed [6]. It was also found that 44% of the mothers had no proper education. This result did not correlate with a study conducted in the Vhembe district of Limpopo Province that showed that 11% of mothers were illiterate, 35% were educated up to grade seven, 52% had a level of education from grade eight to grade 12, and only 2% had a tertiary education [7]. This shows that the literacy rate in Pakistan is still below accepted standards. As a result, stunted and underweight infants were mostly observed in mothers with less education.

More than half of the infants under study were underweight for their age. These factors play an important role in the growth and development of children. Malnutrition is one of the leading health problems in children below the age of five in developing and under-developed countries like India and Bangladesh [8]. Improper weaning practice or delayed weaning is a major cause of malnutrition and thus mothers need to carry out proper weaning practices. In a similar study in Bangladesh, 27.27% of infants between six and 15 months started the weaning process at an age of less than six months, 18.18% started weaning after six months, and 26% of infants started weaning at six months [9]. We also found that more than half of the infants were introduced to semi-solid foods.

In Limpopo, South Africa, however, mothers started weaning with water at less than one month of age in 79.29% of cases and 20.75% started when their children were older than one month. In 36.2% of infants, solids were started at less than one month and 63.8% started with solids when their children were older than one month of age [10]. Considering illness incidences, similar results were seen in a study conducted in Ethiopia where 13.5% of children in the age group of three to 36 months were hospitalized [10]. Children were admitted to the hospital for various reasons including gastroenteritis followed by diarrhea, respiratory disorders, and malnutrition [11]. Underweight children and wasted infants indicate that the consumption of protein and other macronutrients and calorie intake of children is continuously insufficient. This may be related to the poor quality of complementary foods [12].

The current study had a few limitations such as a small sample size due to hesitancy of the general population visiting hospitals to participate in the study and no follow-up of patients. Since the findings are important and interesting, we emphasize the significance of conducting such studies on a larger population that will help in comparing the situation between rural and urban populations as well. Further studies should also be conducted in private hospitals since the majority of the elite and middle class prefers private and personalized treatments to government-based treatment.

## Conclusions

Adequate weaning practices are crucial for the healthy growth and development of infants. From the current study, it can be concluded that the majority of infants were breastfed. However, approximately 56.1% of mothers were poor in their practices for complementary feeding as they started weaning after six months of age. Such poor practices led to 2/3rd of infants being underweight and 1/5th of infants having smaller heights for that age. Similarly, lack of proper weaning education and practices affects the nutrient status of the infants such that it can lead to deficiencies in vitamin A and vitamin D in addition to folate and iron deficiencies. Therefore, mothers should be motivated to start weaning at the appropriate age of six months to minimize nutrient deficiencies. Lack of knowledge among mothers about weaning practices ultimately affects the well-being of infants later in life. Therefore, considerable efforts need to be undertaken to spread awareness among mothers about proper nutrient and weaning requirements of infants to ensure the growing children do not face health problems later in life.

## Appendices

**Table 4:** Macronutrient deficiency

Characteristics	Total (N = 200)
Vitamin A (%)	
Yes	5.5
No	94.5
Vitamin D (%)	
Yes	9.5
No	90.5
Iron (%)	
Yes	3.5
No	96.5

**Table 5:** Association of risk factors and weaning age

		Weaning age			P-value
		<6 months	At 6 months	>6 months	
Diarrhea after weaning	Yes	75.00%	20.80%	4.20%	0.149
	No	54.00%	37.50%	8.50%	
Illness in child	Yes	62.50%	30.70%	6.80%	0.316
	No	51.80%	39.30%	8.90%	
Vitamin D deficiency	Yes	36.80%	52.60%	10.50%	0.0188
	No	58.60%	33.70%	7.70%	
Vitamin A deficiency	Yes	54.50%	36.40%	9.10%	0.0986
	No	56.60%	35.40%	7.90%	
Iron deficiency	Yes	57.10%	42.90%	0%	0.0708
	No	56.60%	35.20%	8.30%	
